# A Pragmatic Review to Assist Planning and Practice in Delivering Nutrition Education to Indigenous Youth

**DOI:** 10.3390/nu11030510

**Published:** 2019-02-27

**Authors:** Robin Kagie, Szu-Yu (Nancy) Lin, Mohammad Akhtar Hussain, Sandra C. Thompson

**Affiliations:** Western Australian Centre for Rural Health, The University of Western Australia, 167 Fitzgerald St, Geraldton, WA 6530, Australia; robin.kagie@uwa.edu.au (R.K.); nancy.lin@uwa.edu.au (S.-Y.N.L.); akhtar.hussain@uwa.edu.au (M.A.H.)

**Keywords:** nutrition, nutrition education, Indigenous, Aboriginal, youth, nutrition tools, school, community, cultural competence, sports, best practices

## Abstract

Many health promotion campaigns have incorporated multi-component nutrition interventions to promote healthy diet-related behaviours among Indigenous communities, particularly children and adolescents. However, these campaigns show mixed results and while research often describes outcomes of approaches and interventions, it does not extensively describe implementation processes and best practices for nutrition education for Indigenous youth. To enhance knowledge and understanding of best processes in nutritional education approaches with Indigenous youth, we conducted a search using multiple databases including PubMed, Google Scholar, the Australian Indigenous HealthInfoNet and Australian government research databases to identify relevant peer-reviewed and grey literature as well as educational resources, such as websites and handbooks for teachers, parents, and students. We list and describe common features of successful nutritional interventions in Indigenous settings, steps for nutrition education targeting youth, school-based nutrition education for different ages, and general guidelines for teaching Indigenous students. Current best practice and knowledge gaps for the delivery of nutrition education to Indigenous youth are described.

## 1. Introduction

The health of Indigenous people across many countries is known to be poorer than that of the wider population [1,2,3]. One impact of colonisation was the disruption of traditional lifestyles, and the exposure to Western diets has resulted in high rates of chronic disease. Our interest pertains to how nutrition education of Indigenous young people could form one component of a multilevel intervention to improve understanding of health and healthy behaviours. While our interest and the focus of this paper is on Indigenous Australians, the findings are likely to have relevance to other Indigenous populations in the developed world as well.

The health disparities experienced by Aboriginal and Torres Strait Islander (hereafter respectfully referred to as Indigenous) Australians are well described, with a life expectancy gap of around 10 years [4]. Australia’s Indigenous peoples represent 3.3% of the national population and live in both cities and rural communities in all states. The median age is 22.3 years compared to 37.0 years for non-Indigenous Australians, which reflects their higher mortality rate [5]. Indigenous Australians are more likely to have diabetes, cancer, and cardiovascular conditions than non-Indigenous Australians, and more strikingly, almost one-fifth of the burden of disease in Aboriginal Australians is attributable to poor nutritional intake [6]. While a more sophisticated understanding of health disparities requires approaches that go beyond exhortations to adopt health behaviours, traditional risk factors such as nutrition and physical activity remain important mediators of poorer health [7].

Poor health in later life is now understood to reflect exposures in developmental stages and early life, including infancy, childhood, pre-adolescence, and adolescence [8,9,10], with the early years having a major and formative influence on patterns of behaviour and ways of thinking. Adversity with an onset during early childhood often continues throughout life and reflects the interplay of social, economic, political, cultural, and environmental factors [11]. Youth are particularly vulnerable to the legacy of colonisation and cultural oppression with resultant intergenerational trauma, which can be manifest as negative behaviours such as high rates of drug and alcohol addiction, violence towards themselves and others, criminal behaviour and interaction with the justice system, homelessness and leaving school early [12].

Questions remain about how to most effectively intervene to reduce the diet-related disparities experienced by Indigenous Australians, recognising that dietary patterns are often influenced by food insecurity, availability, and access issues, and poor socio-economic status. These influence the uptake of non-traditional westernised foods, which are energy-dense and high in saturated fat, salt, and sugars [13]. Early life is a particularly important time for good nutrition because childhood and adolescence are periods of growth and high demand for both calories and nutrients. In addition, eating patterns and food preferences are established early, and there is good evidence linking poor nutrition with higher risks for developing chronic diseases such as cardiovascular disease, hypertension, type II diabetes, and obesity later in life. Earlier onset of underlying abnormalities and disease processes increases risks for dying prematurely [14]. The health disparities outlined above and the importance of nutrition in early life indicate the importance of directing effective nutrition interventions at Indigenous youth, a group known to have suboptimal food and nutritional intake [15].

Many health promotion activities have incorporated multi-component nutrition interventions to promote healthy diet-related behaviours among Indigenous communities, particularly among children and adolescents. However, these interventions have shown mixed results [4,16,17], including programs that are successful in one community or region not necessarily translating to success in a different community or region [17,18,19]. In addition, the evaluation processes for these programs may focus on short-term quantitative dietary outcomes, without offering solutions that address the deeply entrenched societal disparities experienced by Indigenous communities. However, food-supply policies in the settings and efforts to develop Indigenous workforce capacity in nutrition have been identified as effective strategies [17,18,19]. Evidence of the extent to which nutrition-focused campaigns can improve or delay the onset of chronic disease outcomes is limited. Despite this, lifestyle programs that focus on individuals, families, and communities can sustain positive health effects up to three years post intervention [20].

Increasingly, Indigenous health organisations are promoting strength-based approaches, which utilise community resources, knowledge and capacities to foster improvements in the well-being of Indigenous Australians [11]. Recent evidence suggests that community empowerment and local ownership over health promotion programs are two of many critical elements in determining program sustainability and success [21,22]. Engaging local people as experts in program delivery will also help to ensure programs are more suitable for the widely different contexts in which Indigenous people live, in terms of geography, climate, socioeconomic circumstances, food availability and food security. Culturally appropriate programs have been designed to empower children and adolescents to motivate behavioural changes when interacting with other peers [23]. This suggests the need to view children and adolescents as the promoters of health in their immediate surroundings.

This pragmatic review discusses current knowledge on nutrition education, especially in relation to Indigenous Australian youth. We aim to describe information that informs best practice approaches for a comprehensive approach to nutrition education in Indigenous youth. Such information will be valuable to educators, such as those delivering health promotion interventions and school teachers, as it informs implementation of appropriate strategies for nutrition education that will most likely to be effective for young Indigenous populations.

## 2. Methods

Our approach best fits the typology of a pragmatic review, which provides a broad summation of a topic area of value for those coming to the subject for the first time [24]. We undertook the review because we often work with Indigenous Australian youth where there are opportunities to deliver some educational programs, and were interested in how the literature could inform our approach in delivery of education. To identify relevant literature, we focused on peer-reviewed and grey literature, and on websites designed for the public and teachers. Research articles were initially identified in January 2018 through a multiple database search, including PubMed, Google Scholar, the Australian Indigenous HealthInfoNet and Australian government research databases. The relevant literature was identified using search terms that included combinations of nutrition, education, youth, community, health literacy, healthy eating, Indigenous or Aboriginal, and various settings (such as schools or sports clubs). Citation snowballing was used to identify relevant academic and grey literature. Grey literature was also sought on government and other relevant websites such as VicHealth (the Victorian Health Promotion Foundation), and websites that provide resources for teachers, parents and other carers relevant to nutrition education. This approach was adopted recognising that academic research describes outcomes of approaches and interventions, but often does not extensively describe implementation processes and best practices for nutrition education in Indigenous youth. Articles that did not describe the process of implementation or the tools being used were excluded. We included studies undertaken in Canada, the United States of America and Australia because the research is relevant to Indigenous and Native populations in a Westernised environment.

## 3. Findings

### 3.1. Existing Evidence and Principles

The principles identified in the Ottawa Charter (1986) [25] remain relevant to the design and implementation of contemporary nutrition interventions. Successful interventions across the United States (US), Canada, and Australia that have improved community health outcomes have included multiple components that incorporate the following elements in their program: effective assessment and feedback, addressing community priorities, addressing both individual and community issues, long-term partnerships, community engagement and capacity building, cultural competence, multi-level programs, involving respected Indigenous educators, involving Indigenous Community Controlled Organisations, integration with existing services, and involving dedicated, appropriately trained workforce [16,17,18,26,27,28,29] (Table 1). Interventions targeting nutrition, diet, and/or exercise with these features were more likely to have long-term effects, although to date there is no evidence that confirms sustainability for more than five years [18]. Difficulties experienced with undertaking evaluation of programs in these settings have been described [29], as has differential reporting bias based upon self-reporting of dietary intake and store purchase data in 20 Indigenous remote communities [30].

#### 3.1.1. Settings for Nutrition Interventions

A settings-based approach is commonly used when it comes to implementing nutrition programs to encourage healthy eating among Australian youth. Three major categories for settings are recognised; schools, sporting clubs and in the community.

##### Schools

Nutrition interventions are often implemented within schools as these settings serve as important places to influence dietary and behavioural change among children and adolescents. Children who learn about healthy nutrition in school can be change agents that influence the purchase and consumption of healthy foods by their parents [31,32]; the idea of children as health change agents has been described in rural Kenya and Tanzania and highlights the potential of this approach. A school is a convenient setting to implement peer-led interventions as many interactions take place between school children. Examples of nutrition interventions in school settings with Indigenous students include the implementation of nutrition education in the curriculum [18], the introduction or improvement of breakfast/lunch programs [18,33], the introduction of cooking classes [22], culturally appropriate physical activities [16], and a change in provisions of the school canteen [34], which may supplement general healthy nutrition programs such as the National Healthy School Canteen (NHSC) guidelines and resources, state-level Healthy Food and Drink policies and other initiatives like Crunch ’n Sip which are run in schools [33,35,36].

Children can also have a powerful influence on the knowledge and understanding of other children and some school-based interventions have used children as change agents among their peers and to influence their family [37]. A number of studies that have utilised peer-led interventions to educate Australian Indigenous youth on sensitive health topics, resulting in improved behavioural outcomes and an increase in knowledge [23]. Although few studies describe the approach of children as change agents or peer-led interventions for Australian Indigenous communities, those studies that exist on peer-led health interventions in Australian Indigenous youth show evidence of changes in knowledge and attitudes. However, there is limited knowledge on the mechanisms under which peer-led health interventions are effective, suggesting a need for more research and evaluation of projects in this area [23].

##### Sporting Clubs and Facilities

Sports clubs are another area suitable to implement nutrition interventions targeting Indigenous children and adolescents. The pilot study of Reilly et al. (2011) describes the local sports club as a place where locals engage in social events and play sports [34,38]. Interventions in sports facilities include the organisation of nutrition workshops, provision of healthy foods for club members, and improving the nutritional quality of canteen provisions [17,34,38]. Although not all Indigenous people like sports and are good at sports [39], sport does help bring Indigenous children and families together [40].

Australian health promotion foundations such as Healthway and VicHealth have provided Healthy Club Sponsorships to sporting clubs to develop and implement sponsorship around health-promoting environments within sporting clubs targeting understanding and healthy behaviours in areas such as nutrition, smoking and being Sunsmart [41,42]. While such an approach to influencing community norms and the availability of healthy foods is likely to be important in the longer term, the direct efficiency of such healthy club sponsorships is difficult to assess, although it is considered to have importance for clubs regarding rejecting junk food sponsorship [43].

##### Communities

The implementation of documented interventions to improve access to healthy foods and improve nutrition, and which operate at the Indigenous community level, have occurred over many decades. Numerous interventions have been implemented since the 1980s, including providing and promoting healthy nutrition in remote community stores, increasing the transportation of fresh produce, and implementing rules and regulations that restrict access to unhealthy foods [34]. Fruit and vegetable subsidies, cooking classes, return to traditional lifestyles and preschool programs have often included an educational component included as part of the intervention [44,45,46,47,48], however, it is difficult from the reports to assess the nature and content of the education component and the target age group was often unspecified.

### 3.2. Health Literacy and Nutrition Education

The development of nutrition education programs cannot proceed without taking health literacy into account. To be able to educate any population about nutrition, those delivering the program need to ensure the information they provide can be understood. Vass et al. described a situation in which their target population did not recognise certain biomedical health concepts as part of their worldview and had no words for these concepts in their language. It appeared that the concept of nutrients being broken down inside the body for uptake in the blood circulation was not known as such in the worldview of the Yolŋu people [3]. Another situation in which health literacy greatly influences nutrition education is when program recipients are illiterate and thus unable to read labels on food packaging [49]. Many of the nutrition education guides that exist, such as the “Healthy Canteen Kit—Student Learning Activities” guide [50], have a component that focuses on learning how to pick the right foods by looking at their ingredient lists, an approach that poses difficulties for illiterate individuals. The traffic light system, which is also used by the “Healthy Canteen Kit” and other government programs, is another way to categorise food, using green for “everyday” foods, amber for “select carefully” foods, and red for “occasional” foods, but even this tool might be problematic for remote communities where road traffic lights do not exist. There are other tools that can aid in delivering nutrition education, such as the Australian guide to healthy eating from the Northern Territory, which has an adapted version named “The Aboriginal and Torres Strait Islander guide to healthy eating” [51]. This food wheel includes foods that are commonly eaten in rural and remote areas, such as kangaroo and damper. However, Indigenous people do not only live in rural and remote areas and such an educational resource still needs to be incorporated into more comprehensive nutrition education. Since health literacy levels greatly impact the ability of an individual to make healthy (food) choices, increasing health literacy and nutrition education go hand in hand. Moreover, health education and communication combined, determine the level of health literacy [52]. Low health literacy limits the success of nutrition education, although there is evidence that nutrition education can work. A study of attendees at a FOODcents^®^ adult nutrition education in Western Australia with a high proportion of Indigenous people (169/875 = 19%) found that perceptions of course usefulness were high among Indigenous and non-Indigenous participants, and that there were significantly larger improvements in confidence, nutrition knowledge, and reported consumption behaviours in Indigenous participants [53].

### 3.3. Engaging with Indigenous Australian Youth

As a community participatory-based approach, youth-led programs represent an emerging field for addressing the health needs of young individuals in Australia. Youth empowerment and engagement strategies have emerged to improve health outcomes [54].

A key factor in Indigenous health education and interventions is for the recipients to be able to relate to the material presented [55]. There have been several interventions targeting Australian Indigenous youth that aim to make education sessions interactive, audio-visual, and hands-on. Implementers have found various ways to make health education interactive, ranging from video messages recorded by Indigenous elders to bush camps and rap songs [56]. Interventions, like rap song projects, seem to have a positive effect on health outcomes on the short term, but there is no evidence they are sustained in the long-term, after the funding stops. Moreover, the impact on health behaviour is unclear.

#### 3.3.1. What Works in Delivering Nutrition Education to Youth?

Despite much of the relevant literature on the subject of nutrition interventions referring to “education”, very few studies describe the actual process of educational intervention initiation, implementation, and institutionalisation. Lack of detailed information about processes for education and health communication makes it difficult to readily implement effective evidence-based programs. Here, we explore underlying principles, practice and lessons for effective educational interventions related to nutrition that could make it easier for others planning and delivering youth-focused education.

##### Implementation Process

Various studies have described steps for implementing nutrition education targeting youth (Table 2). A study performed in New York City interviewed staff and volunteers from several schools about the implementation of nutrition education programs they had run in their school [57]. The study discovered four domains that play an important role in implementing nutrition education programs: building motivation; choosing programs; developing capacity; and legitimising nutrition education. An important aspect in the description of these domains was how well a program fits within a certain school. Ultimately, each school decided what nutrition program they wanted to implement in their school and this depended on the organisational structure of the school, the school’s aims, and the suitability of the program across different levels [57]. This study offers a valuable insight into the need to tailor nutrition education program delivery to the school setting and level, and can inform guidelines for program delivery. No similar studies describing in detail the processes of nutrition education program implementation in Australian schools with or without Indigenous students were identified.

The U.S. Department of Health has described the steps to make a health communication program work [58]. The “Pink Book” book serves as a guideline for public messaging that is much broader than nutrition education in schools, although several components described can be directly translated to the stages of the nutrition education program implementation. The book describes four stages that run in a continuous loop. The first stage is “Planning and Strategy Development” and focuses on learning about your audience, exploring the setting and exploring various internal channels, identifying partners, assigning responsibilities, and thinking of ways to communicate information. The second stage, “Developing and Pretesting Concepts, Messages, and Materials”, focuses on determining approaches and methods to fit the audience, culturally appropriate messages and materials, choosing messages for sensation-seeking youth, the appeal of a message (humorous, emotional, shocking), what kind of material fits for certain outlets, and pretesting of materials. The third stage, “Implementing the Program”, focuses on process evaluation and partnership communication throughout the program. The fourth stage, “Assessing Effectiveness and Making Refinements”, focuses on outcome evaluation and revision [58].

A similar guide was published in 2017 by the United States Agency for International Development [59]. They present a road-map consisting of five steps: (1) assess needs, (2) design strategy, (3) create and iterate, (4) mobilize and monitor, and (5) evaluate and evolve. These steps address very similar factors to the stages described in the Pink Book.

Another way to engage with youth is via phone applications, websites, and other technologies. The U.S. Department of Agriculture’s Food and Nutrition service developed a nutrition program provider called “Team Nutrition”. Team Nutrition offers nutrition education materials for incorporation into the school curriculum with lessons that can be integrated into maths, language, arts, and science [60]. These materials include a teacher’s guide, a student workbook, and parent handouts. In addition, it refers to a website/food guide called MyPlate, introduced in 2010, which educates children and adolescents through games, interactive and creative assignments, and easy-to-read information pages [61].

One of the “best practices” for nutrition education programs in the U.S., described by Baker et al., used apps during an intervention for elementary-aged youth [62]. The “Body Quest: Food of the Warrior” program is a childhood obesity prevention initiative that involves an iPad application that supplements classroom education and utilises animated warrior characters as role models for the students. The program had been running for three years in 2014 and was considered successful enough to then become fully funded by the federal government [63].

In another project described by Baker et al. (2014), educator observations were emphasised to ensure that the program is being delivered as designed. Observing educators increases fidelity, which is of key importance when a program is developed for a very specific audience [62]. Another section focused on the methods used by educators. When the educators knew where their (future) students did their shopping and undertook exercise, they could tailor the program delivery and increase the relevancy. Thus, where foods were on sale could be aligned with nutrition education [62], and seasonal fruit and vegetables from the school garden could be used for nutrition education in class [57].

The value of collaborations in program delivery was highlighted and a particular collaboration, which emerged between a school and a Radio Disney station, was described. A presentation on nutrition and physical activity was held in class with participants receiving Disney items if they had attended [62].

##### School Curriculum Interventions

Schools are where Australian children spend around 6 hours per weekday in every school term so make an ideal location in which to deliver youth-focused nutrition education. Each state in Australia has published one or multiple guides, informed by nutrition studies, that instruct school canteens on which healthy foods to provide and how to engage students in the process of making healthy choices. The “Healthy Canteen Kit” series by the Victorian State Government is one example. This guide uses a traffic light system to categorise food into bad, better, and healthy choices [64]. Some of these guides provide examples of nutrition education for students, such as the “Healthy Canteen Kit—Student Learning Activities” guide shown in Table 3 [50]. This resource suggests several activities for students in Levels 1 to 6 and addresses the key elements of the Victorian essential learning standards. Indigenous students are not specifically addressed and the guide does not include cultural elements such as traditional foods and preparation methods. Such resources may need to be adapted to be suitable for education of various population subgroups. For example, various kitchen tools are mentioned in the guide, yet students from disadvantaged families (Indigenous and non-Indigenous) may not have access to the implements available in the homes of children in more advantaged circumstances [65]. The guide also assumes that Level 6 children can read and write, which is not the case for all Level 6 students. Appreciating the fact that Australian schools measure student performance according to western standards that do not harmonise with Indigenous culture and learning styles, this assumption might not be appropriate for Indigenous students either [66]. Despite the guide not being tailored specifically for use within schools with Indigenous students, it still includes information on ways to deliver nutrition education that might be valuable for these schools.

##### Delivering Education to Indigenous People

Delivering education to Indigenous people requires an approach that is culturally appropriate. According to Stephen Harris (1984), known for the development and popularisation of the ‘Aboriginal Learning Styles’ theory, this approach is different from the approach towards mainstream Australians [67,68]. Harris explains that Indigenous students learn by observation and imitation, trial and error, acting in real life, developing task-specific skills, and focusing on people and relationships [68]. However, this approach has been criticised by Watt and colleagues because it implies that the problem lies with suggested incapability of Indigenous students to be able to engage in structured, theoretical learning [68]. According to Watt, this is not true, as within Indigenous culture there are ceremonies and practices that involve this learning style. Martin and colleagues also challenge the theory of Indigenous learning styles, as there is little empirical evidence to support the notion that these learning styles improve school achievements for Indigenous students [69]. Nakata, an Indigenous researcher and co-author of the article, has commented on the lack of evidence to support the success of Indigenous learning styles and authored several articles that describe the dangers of trying to solve the mismatches in class by adopting Indigenous learning styles [58]. Canadian researcher Ledoux has also discussed the concept of Indigenous learning styles and raised several points made by various authors, such as generalisation and stereotyping, pointing out that these generalisations reflect important aspects of Indigenous culture that impact knowledge and pedagogy [70]. Ledoux suggested the following general guidelines for teaching Indigenous students (Table 4).

Despite the subject of Indigenous learning styles being contested, the general guidelines as described by Ledoux seem useful to consider during the development and implementation of education for Indigenous people.

#### 3.3.2. What Does Not Work in Delivering Nutrition Education to Indigenous Youth?

Interventions that do not specifically take the Indigenous cultural background into account tend to be less successful and less sustainable [16]. Antonio et al. (2015) describe a nutrition intervention that was considered culturally competent because it had tailored the intervention according to the youth’s environment and everyday experiences, but had not incorporated cultural practices. This intervention proved to be unsuccessful in improving health outcomes in Native American adolescents [16], indicating that just tailoring an intervention to match the environment and experiences might not be enough. In addition to lifestyle interventions that are not culturally competent generally being unsuccessful in improving health outcomes, many interventions that appear to be successful in the short-term are not sustained. Often there is a limited period of follow-up, rarely beyond three years, which limits the evidence for program sustainability beyond program duration [20]. Indigenous youth can be avid uses of modern technology such as the internet and social media, and there is little doubt about the growth in use and potential of this as a means for education of this group [71]. However, to date there has not been evidence of successful health promotion interventions using social media and mobile apps in this group [72], although this is likely to be an important area for research going forward. Lack of availability of healthy foods in stores is another complication that often leads to unsuccessful nutrition education programs [4,34]. With the proper knowledge about healthy food, but without healthy food choices readily available in community stores, any educational intervention will be unlikely to lead to improved outcomes or be sustainable.

Community involvement is yet another important factor that has been reported to greatly impact on whether an intervention proves sustainable [16,21]. Capacity building, participatory decision-making, learning by doing, the use of local cultural and ecosystem knowledge and resources, and networking are key factors for success [55]. Table 1 shows that community ownership is one of the common features that lead to a successful intervention, and that it increases program sustainability. Although studies ensuring community involvement have not achieved long-term sustainability either, studies not involving the community have lower sustainability of outcomes [16].

## 4. Conclusions

Many of those with roles working with Indigenous youth have opportunities to provide education that could affect this group’s understanding and readiness to adopt healthier lifestyle behaviours. This occurs in many settings, so the purpose of this pragmatic review was to assist in identifying principles, approaches and resources that could inform nutrition education opportunities. While general guides exist and some Indigenous specific resources, there was no overall guidance for working in this area with Indigenous youth.

The challenge of program sustainability was identified by numerous authors of papers on nutrition education. However, the authors were unable to identify the existence of a protocol or guide to assist those interested in delivering nutrition education to Indigenous Australian youth, either at the community or school level. The state-specific guides, like the Victorian “Healthy Canteen Kit” series, focus on nutrition interventions in schools only and do not distinguish Indigenous students’ needs. Moreover, the guides (and associated programs) mainly target canteen provisions, dedicating only a small proportion of their information to nutrition education. Such educational programs have been developed as part of trials of obesity prevention with American Indian children in the US as part of a multi-faceted interventions, with the investigators emphasising Indigenous learning modes that included learning through observation and practice, learning from storytelling, learning metaphorically, holistic learning, learning by trial and error, learning through play, learning cooperatively, and learning through reflection [73].

Although the recommended step-wise approaches to health communication presented in Table 2 and Table 3 are not specific to either Indigenous people or youth, they offer a useful approach to health education and communication that is easy to follow. Step-wise approaches such as these could prove valuable if tailored to target nutrition education in Indigenous youth. However, as it is now, the efficacy of a step-wise approach to implementing nutrition education for Indigenous youth in terms of engagement and outcomes is unknown. We are also aware that train-the-trainer has been an effective approach in some health promotion interventions [74], including those related to nutrition [75], although relevant articles were not identified under our search strategy.

This pragmatic review intends to inform practitioners who are not nutrition or even education experts about approaches to consider when working with Indigenous youth where a health promotion intervention should include a nutritional education component. Several health interventions in Indigenous communities were identified, but none comprehensively described the implementation process of nutrition education for Indigenous Australian youth. Moreover, while there are papers that report on principles and features of best practice programs, there has been no systematic assessment of methods that work for the delivery of nutrition education for Indigenous youth, making this area an evidence gap that warrants further attention. We recommend that future research should report in more detail the approach and detail of the “what and how” by which nutrition education is delivered when working with Indigenous youth. Given the importance of education as a means of increasing health literacy, even within the context of multifaceted interventions, more focused attention on education as an important component of nutritional interventions is needed.

## Figures and Tables

**Table 1:** Common features of successful nutrition interventions.

Feature	Explanation
Effective assessment and feedback	Program assessment and feedback to individuals participating in the program
Addressing community priorities	Focus on community issues and not the goals of the program deliverer
Addressing both individual and community issues	Support for healthy nutrition through cooking classes, subsidising meals, improving healthy food supply
Long-term partnerships	Sustain commitment between the program leaders and the community
Community engagement and capacity building	Involve the community in the program and giving them ownership over the program to ensure sustainability
Cultural competence	Utilise curricula based on Indigenous culture, with culturally appropriate support and methods like story-telling and cultural dancing in physical exercise
Multi-level programs	Offer nutrition education (Level 1) and modify the environment to become more healthy-food friendly by increasing accessibility to healthy foods (Level 2)
Involving respected Indigenous educators	Have community elders direct the program, employ Indigenous staff, and ensure the support of key community members
Involving Indigenous Community Controlled Organisations	Base programs where Indigenous people attend for health services
Integration with existing services	Add nutrition education to the school curriculum and/or maternal and child health care services
Involving dedicated, appropriately trained workforce	Create dedicated positions for Aboriginal and Torres Strait Islander people and value their contributions

**Table 2:** Steps for nutrition education targeting youth, adapted from Porter et al. (2018), the U.S. Department of Health (1992) and Salem et al. (2017) [56,57,58,59].

Step	Explanation
**1. Orientation**	Who is the audience? What are their interests? What is their daily schedule? What kind of activities do they do? What is their background? What is their behaviour? What is the (school) setting? Where is it located? What are the facilities? What is the environment like? What are the rules, regulations, and principles within the setting?
**2. Methodology**	Levels of implementation Youth within the entire setting or certain age groups/grades Appropriate materials Digital (apps/videos/computer), interactive, narratives, activities (physical activity, gardening activity, games) Cultural components (cultural dance in physical activity, Indigenous elders in videos/narratives) Appeal of educational messages Humorous approach Emotional approach Shocking approach Setting goals and developing guidelines Assigning tasks and responsibilities Creating partnerships Local grocery stores Swimming pool Indigenous organisations and elders
**3. Creation**	Create suitable methods for the specific audience and setting Develop culturally appropriate and relevant methods Fitting program within existing curriculum/regulations/values Test-run the program Do the partnerships work? Are the responsibilities being managed? Is it culturally appropriate? Is the program appropriate for the setting/environment/target group? Is it well-received? Does it have potential?
**4. Execution**	Intervention implementation Continuing evaluation and feedback Maintain communication Maintain partnerships
**5. Evaluation and Refinement**	Evaluate entire program Refine/terminate the program according to feedback Documentation of program implementation processes

**Table 3:** Several examples of nutrition education from the “Healthy Canteen Kit—Student Learning Activities” [52].

**Level 1**	What foods do you eat? (conversation teacher–students) Grouping foods exercise (categorising pictures of food) Safety rules (making a poster on actions before handling food e.g., hand-washing) Taste testing (blindfolding students and have them taste/smell/feel foods) Harrison the Healthy Bear * (students take the class teddy home and explain the next day what he had for dinner, etc.) Weekly Lunch box (one student a week presents a lunch idea with healthy foods)
**Level 2**	Why do we eat? (conversation teacher–students) Food categorisation (grouping into Everyday, Select Carefully, and Occasional) Food groups (putting categorised foods in a pyramid and discussing the pyramid–worksheet available) The very hungry caterpillar (reading a story based on food categorisation) Fruity kebabs (constructing a fruity kebab using safety rules and taste-testing the components)
**Level 3**	The why, what and where about eating lunch (brainstorming on reasons why to eat lunch, using sticky notes, group discussion) Lunch in the past (discussing what ancestors ate for lunch, compare parents’ lunch with own lunch) Eating at the canteen (categorising canteen foods into Everyday, Select Carefully and Occasionally) Ordering lunch (composing a $5,- lunch and discussing the choices among the group) Food handling skills (how to use tongs, gloves, knives, etc. -> students make a video or photograph presentation) Creating quick and easy healthy lunches (making a shopping list for healthy recipes)
**Level 4**	Investigating food options (what foods should be eaten in what proportions) Investigating design options (what foods could be the wrapper of the wrap, what foods could be inside a wrap–designing and discussing) Promoting the wrap (making a poster, making the wrap and photographing it, presenting the wrap) Evaluating the wrap (receiving feedback on the wrap and re-designing)
**Level 5**	Investigating healthy food choices currently available at the canteen/shop (categorising foods into Everyday, Select Carefully, and Occasionally and discussing these in class) Factors that influence students’ food choices (making a chart with these factors such as cost, hunger, time, etc.) Design brief and production: ’Meal Deal’ (developing a meal that can be sold for lunch at the canteen/shop) Promoting the ‘Meal Deal’ (making a poster/pamphlet on the computer to promote the ‘Meal Deal’ with its nutrient content, taste, etc.)
**Level 6**	Thinking about food choices (what influences meal choices–class discussion) What’s on the label (matching the ingredient lists to foods) What’s that I’m eating? (discussing the food labels of foods sold at the canteen/shop) Cooking challenge (designing an Everyday category sandwich, roll, or wrap and producing it) Designing a healthy lunch pack (designing a healthy lunch which will be judged by a panel–winning lunch will be sold in the canteen) Promoting healthy choices (promoting a lunch for a target group, such as a picnic lunch for the Level 1 kids–taking care of each other)

* The bear can be renamed by the students. Note: Some examples have been excluded from this table because they assume that students use lunch boxes, possess a variety of kitchen tools at home, or have magazines/brochures available and might not be applicable in certain (low-income) settings [65].

**Table 4:** General Guidelines for Teaching Indigenous Students cited from Ledoux (2006) [70].

Learners should be given an opportunity to privately rehearse a skill before demonstrating competency publicly Individual learners should not be spotlighted Warmer, more personal teaching styles are most effective Silences and longer pauses after asking questions are to be expected An overview of the lesson is the best way to begin Desired behaviours should be reinforced indirectly rather than by using direct praise Sensitivity to non-verbal cues is necessary More global, holistic instructional approaches which emphasise the development of self-esteem, confidence, and empowerment are desirable
